# Effects of Normoxic Recovery on Intima-Media Thickness of Aorta and Pulmonary Artery Following Intermittent Hypoxia in Mice

**DOI:** 10.3389/fphys.2020.583735

**Published:** 2020-10-22

**Authors:** Akira Umeda, Kazuya Miyagawa, Atsumi Mochida, Hiroshi Takeda, Kotaro Takeda, Yasumasa Okada, David Gozal

**Affiliations:** ^1^Department of Respiratory Medicine, International University of Health and Welfare Shioya Hospital, Yaita, Japan; ^2^Department of Pharmacology, School of Pharmacy, International University of Health and Welfare, Otawara, Japan; ^3^Faculty of Rehabilitation, School of Healthcare, Fujita Health University, Toyoake, Japan; ^4^Department of Internal Medicine, National Hospital Organization Murayama Medical Center, Musashimurayama, Japan; ^5^Department of Child Health and the Child Health Research Institute, MU Women’s and Children’s Hospital, University of Missouri, Columbia, MO, United States

**Keywords:** intermittent hypoxia, aorta, pulmonary artery, mouse, sleep apnea syndrome, intima-media thickness

## Abstract

Obstructive sleep apnea (OSA) patients are at risk for increased blood pressure and carotid intima-media thickness (IMT), with pulmonary hypertension and right-sided heart failure potentially developing as well. Chronic intermittent hypoxia (IH) has been used as an OSA model in animals, but its effects on vascular beds have not been evaluated using objective unbiased tools. Previously published and current experimental data in mice exposed to IH were evaluated for IMT in aorta and pulmonary artery (PA) after IH with or without normoxic recovery using software for meta-analysis, Review Manager 5. Because IMT data reports on PA were extremely scarce, atherosclerotic area percentage from lumen data was also evaluated. IH significantly increased IMT parameters in both aorta and PA as illustrated by Forest plots (*P* < 0.01), which also confirmed that IMT values after normoxic recovery were within the normal range in both vascular beds. One-sided scarce lower areas in Funnel Plots were seen for both aorta and PA indicating the likelihood of significant publication bias. Forest and Funnel plots, which provide unbiased assessments of published and current data, suggest that IH exposures may induce IMT thickening that may be reversed by normoxic recovery in both aorta and PA. In light of the potential likelihood of publication bias, future studies are needed to confirm or refute the findings. In conclusion, OSA may induce IMT thickening (e.g., aorta and/or PA), but the treatment (e.g., nasal continuous positive airway pressure) will likely lead to improvements in such findings.

## Introduction

Obstructive sleep apnea (OSA) and elevated systemic blood pressure tend to co-exist (Davies et al., 2000) and are associated with insulin resistance, dyslipidemia, atherosclerosis and increased risk of ischemic cardiovascular diseases. However, the causal contributions of chronic intermittent hypoxia (IH), one of the hallmarks phenotypic features of OSA, to these cardiovascular and metabolic abnormalities is unclear. Inferential evidence derived from OSA interventional studies indicates that nasal continuous positive airway pressure (CPAP) can improve baroreceptor responsiveness and reduce waking blood pressure (Coughlin et al., 2007). Systemic blood pressure in mice is elevated by chronic IH (Campen et al., 2005; Schulz et al., 2014), with possible mechanisms of OSA-related hypertension including sympathetic overactivity, and up-regulation of the endothelin and renin-angiotensin systems (Narkiewicz and Somers, 1997; Phillips et al., 1999; Møller et al., 2003). In the context of atherosclerosis and vascular dysfunction, increased carotid intima-media thickness (IMT) has been reported in OSA patients (Drager et al., 2005). Subsequent studies revealed independent associations between hypoxic stress and IMT in OSA (Minoguchi et al., 2005) that can be reversed by nasal CPAP (Bradley and Floras, 2009).

Similarly, IH exposures mimicking OSA induce pulmonary hypertension in mice (Fagan et al., 2001; Campen et al., 2005; Haslip et al., 2015), and work by Fagan et al. (2001) in mice showed that IH induced pulmonary vascular remodeling that paralleled the effects seen during chronic sustained hypoxia. Severe OSA patients have been reported to develop chronic pulmonary hypertension and right-sided heart failure in 12–20% (Bradley et al., 1985; Weitzenblum et al., 1988). Medial thickness of the pulmonary artery (PA) is increased by hypoxia in mice (Hales et al., 1983; Wanstall et al., 2002; Matsui et al., 2004; Ambalavanan et al., 2005; Yu et al., 2008; Mizuno et al., 2011) and in rats (Meyrick and Reid, 1980; Hu et al., 1989; Jeffery and Wanstall, 1999). IH combined with episodic hypercapnia accelerates atherosclerosis of PA in ApoE knockout mice and Ldlr knockout mice (Xue et al., 2017; Imamura et al., 2019), as illustrated by increases in the atherosclerotic area percentage of the PA lumen.

Recently, we conducted experiments in C57BL/7J male mice and found that chronic IH did not decrease O_2_ consumption or energy expenditure, but increased the size of visceral adipocytes during normoxic recovery, the latter aimed at exploring the effect of efficacious treatment of OSA (Umeda et al., 2020; Readers are encouraged to download this paper and its supplementary files). Here, we took advantage of existing fixed aorta and PA from these experiments, and evaluated the effects of 8-week IH exposures followed by normoxic recovery on the IMT of aorta and PA. To enable unbiased comparisons of our experimental data with the data originating from published studies, we used the freely available software Review Manager 5 (RevMan 5).

## Materials and Methods

### Animals and Exposures to IH

C57BL/6J male mice (4–5 week old) were assigned to one of 3 exposure profiles with body weight matching, as previously described (Umeda et al., 2020): (a) 12 h/day of mild IH (IHM) consisting of alternating F_I_O_2_-10-11% and normoxia (F_I_O_2_-21%) with a 640 s total cycle duration (*n* = 7); (b) 12 h/day of severe IH (IHS) consisting of alternating F_I_O_2_-6-7% and normoxia, and a 180 s cycle duration) (*n* = 7); (c) sham IH [Control (CO)] with normoxic air every day, with all exposures lasting for 8 weeks (*n* = 7). After completion of exposures, all mice were kept under normoxic conditions for 5 weeks (IUHW animal experiment ethic committee approval number: 18020). An approximate 12-h dark/light cycle was maintained (approximately 18:00–6:00/6:00–18:00, according to natural light). Mice had free access to regular chow and water for the duration of the experiments.

### Histological Analysis

After euthanasia by decapitation at the end of the 5-week normoxic recovery period, organs underwent fixation with 4% paraformaldehyde. The descending aorta and lung were cross-sectioned using paraffin at a thickness of 3 micrometer, and digital photographs were acquired using light microscopy (MCD-350, Olympus, Tokyo, Japan). Hematoxylin and eosin stain and Elastica van Gieson stain were performed. The IMT data of aorta and PA were analyzed by WinROOF software (Mitani Corporation, Tokyo, Japan), by measuring the area of intima-medial layer on the cross sections of descending aorta or PA. The PA with diameter size of approximately 200 μm was selected. Wanstall et al. (2002) reported that the morphological changes of PA medial thickness by hypoxia in mice were more definitively identified when vessels examined had outer diameters of 151–420 μm when compared to pulmonary vessels with diameters ranging from 50 to 150 μm.

### Collection of Published Data

**FIGURE 1:**
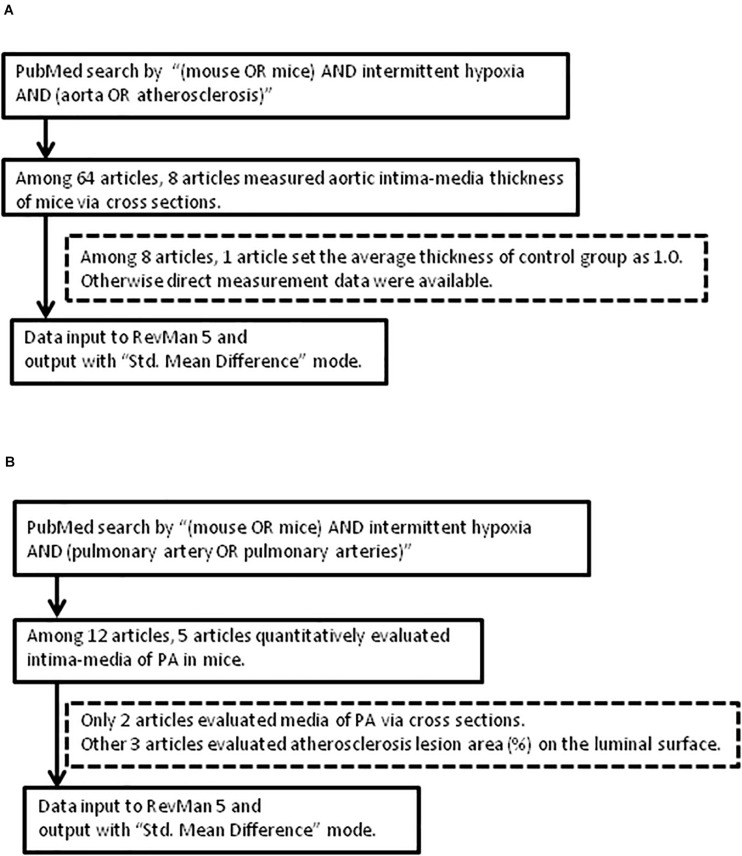
Flow of article selection diagram. **(A)** Aorta. **(B)** Pulmonary artery (PA). RevMan 5: Review Manager 5.

PubMed database was used for the identification and collection of similar experiments with the search commands of “(mouse OR mice) AND intermittent hypoxia AND (aorta OR atherosclerosis)” for aorta, and “(mouse OR mice) AND intermittent hypoxia AND (pulmonary artery OR pulmonary arteries)” for PA. All published articles were then reviewed and relevant studies were retained for analyses (Figure 1).

### Data Analysis

Data are expressed as mean ± standard error of the mean (SEM), unless otherwise indicated. Comparisons among groups were conducted with ANOVA procedures followed by Fisher’s *post hoc* tests. Excel Statistics software, 2010 version (Social Survey Research Information, Co., Ltd., Tokyo, Japan) was used. For comparisons with previous similar experimental data, the free software RevMan 5 (Cochrane) was used. RevMan is the software developed for Cochrane review authors to perform analyses toward a Cochrane systematic review of a healthcare intervention, and this software (currently version 5.3) is available as free software^1^. We undertook the evaluation of experimental data from our own studies and those of previous publications using this software, and uncovered its usefulness for the unbiased overview of relevant experimental data. For all statistical analyses, *P*-value < 0.05 was considered to denote a statistically significant difference.

## Results

### Identification and Collection of Similar Experimental Data

The approach and selection of relevant studies are shown in Figure 1, and ultimately eight articles were found with data on IMT measures of cross-sectional specimens of aorta in mice (Figure 1A and Table 1). In one of these eight articles, the average control IMT was set as an arbitrary value of 1.0. All other articles showed data of IMT measures as micrometers. All the experiments were performed on wild-type mice on aorta (Table 1). However, we found only two articles which evaluated IMT of PA via cross sectional specimens (Figure 1B and Table 2). Because the number of articles on PA IMT measures was very small, we also selected three additional articles which evaluated the atherosclerosis lesion area (%) on the luminal surface of PA. Douglas et al. (2013); Xue et al. (2017), and Imamura et al. (2019) used transgenic mice on PA (Table 2).

**TABLE 1:** Profiles of similar experiments compared on aorta.

**First author (year)**	**Mice**	**Sex**	**Starting age**	***N***	**IH duration (normoxic recovery duration)**	**IH condition**
Dematteis et al., 2008	WT (C57BL/6J)	Male	8 week	4–5/group	14 day (-)	8 h/d, F_I_O_2_-4-21%, 30 s cycle
Arnaud et al., 2011	WT (C57BL/6J)	Male	8 week	7/group	14 day (-)	8 h/d, F_I_O_2_-5-21%, 60 s cycle
Zhou et al., 2014	WT (129S1)	?	Adult	6/group	8 week (-)	12 h/d, F_I_O_2_-8-21%, 30 cycles/h
Poulain et al., 2015	WT (C57BL/6J)	Male	17 week	10/group	4 week (-)	8 h/d, F_I_O2-5-21%, 1 min cycle,
Gras et al., 2016	WT (C57BL/6J)	?	7–9 week	5–6/group	14 day (-)	8 h/d, F_I_O_2_-5-21%, 1 min cycle
Castro-Grattoni et al., 2016	WT (C57BL/6J)	Male	6 week	10/group	6 week (-)	6 h/d, F_I_O_2_-5-21%, 1 min cycle
	WT (C57BL/6J)	Male	6 week	10/group	6 week (6 week)	
Lan et al., 2017	WT (C57BL/6J)	Female	5–8 week	6/group	28 day (-)	8 h/d, F_I_O_2_-5-21%, 40 cycle/h
Arnaud et al., 2018	WT (C57BL/6J)?	?	?	9–13/group	14 day (-)	8 h/d, F_I_O_2_-5-21%, 1 min cycle
Suarez-Giron et al., 2018	WT (C57BL/6J)	Male	6 week	10/group	6 week (-)	6 h/d, F_I_O_2_-5-21%, 60 s cycle
Umeda et al., 2020	WT (C57BL/6J)	Male	4–5 week	7/group	8 week (5 week)	12 h/d, F_I_O_2_-6-7% and normoxia, 180 s cycle

*All the reports measured intima-media thickness of aorta via cross sections. Zhou et al. (2014) set the average thickness of control group as 1.0. Otherwise direct measurements in microns were reported. WT, wild-type mice.*

**TABLE 2:** Profiles of similar experiments compared on pulmonary artery.

**First author (year)**	**Mice**	**Sex**	**Starting age**	***N***	**Chow**	**IH duration (Normoxic recovery duration)**	**IH condition**
Douglas et al., 2013	Ldlr	Male	2–3 month	CO 4, IH 4	HFD	8 week (-)	10 h/d. 4 min F_I_O_2_-8%, F_I_CO_2_-8%/4 min normoxia F_I_O_2_-21%, F_I_CO_2_-0.1%, Ramp intervals of 1–2 min.
	Ldlr	Male	2–3 month	4/group?	Regular	8 week (-)	
Xue et al., 2017	ApoE	Male	10 week	CO 4, IH 8	HFD	8 week (-)	10 h/d, 4 min F_I_O_2_-8%, F_I_CO_2_-8%/4 min normoxia F_I_O_2_-21%, F_I_CO_2_-0.1%, Ramp intervals of 1–2 min.
	Ldlr	Male	10 week	CO 7, IH 8	HFD	8 week (-)	
Imamura et al., 2019	Ldlr	Male	?	CO 7, IH 6	HFD	8 week (-)	10 h/d, F_I_O_2_-8-21%, F_I_CO_2_ (0.5–8) 4 min alternating (1–2 min Ramp intervals)
Fu et al., 2020	WT (C57BL/6J)	Male	6 week	5/group	Regular	6 week (-)	9 h/d, F_I_O_2_-5-21%, 1 min cycle.
Song et al., 2020	WT (C57BL/6J)	Male	8 week	6/group	Regular (?)	4 week (-)	8 h/d, F_I_O_2_-4-21%, 1 min cycle.
Umeda et al., 2020	WT (C57BL/6J)	Male	4–5 week	7/group	Regular	8 week (5 week)	12 h/d, F_I_O_2_-6-7% and normoxia, 180 s cycle

*Only Fu et al. (2020) and Song et al. (2020), and we evaluated intima-media thickness of pulmonary arteries via cross sections. Other three articles evaluated atherosclerosis lesion area (%) on the luminal surface. ApoE, ApoE knockout mice; CO, control; HFD, high fat diet; IH, intermittent hypoxia; Ldlr, Ldlr knockout mice; WT, wild-type mice.*

### Histological Analysis

**FIGURE 2:**
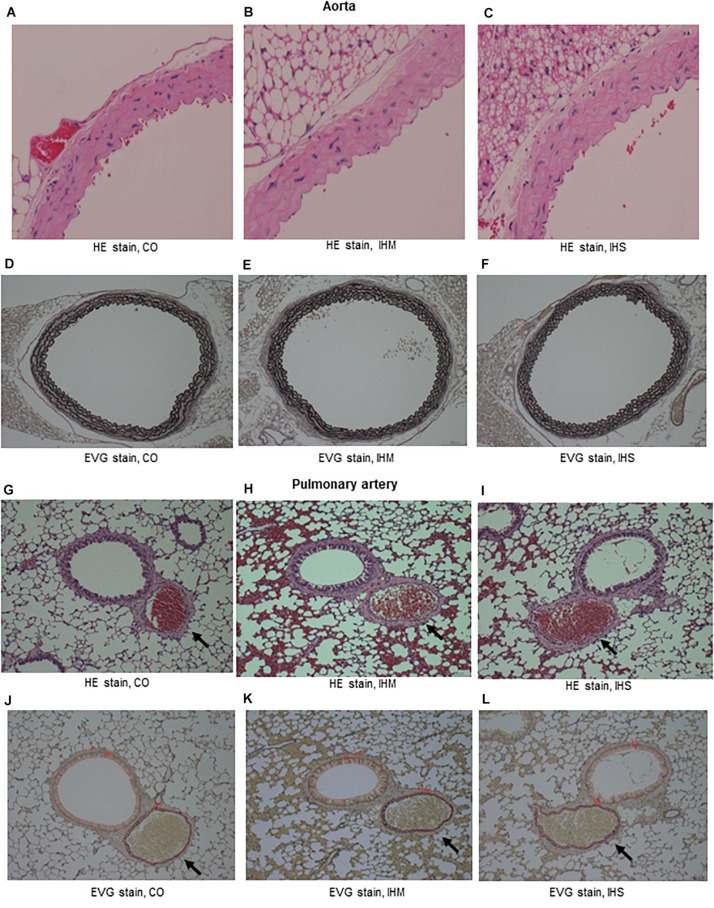
Microphotographs of cross-sectional specimens. **(A–C)** Aorta, Hematoxylin and eosin (HE) stain, initial magnification: x40. **(D–F)** Aorta, Elastica van Gieson stain (EVG) stain, initial magnification: x10. **(G–I)** Pulmonary artery (PA, arrows), HE stain, initial magnification: x10. **(J–L)** PA (arrows), EVG stain, initial magnification: x10. Intima-media thickness of PA is under measure using WinROOF software. Thickness of bronchial epithelium is also measured (data not shown). CO, control; IHM, mild intermittent hypoxia; IHS, severe intermittent hypoxia. Neither IHS nor IHM induced significant changes in intima-media thickness of aorta or PA in the normoxic recovery phase. Persistent disruptions of the elastic laminae were not clearly seen for aorta or PA.

**FIGURE 3:**
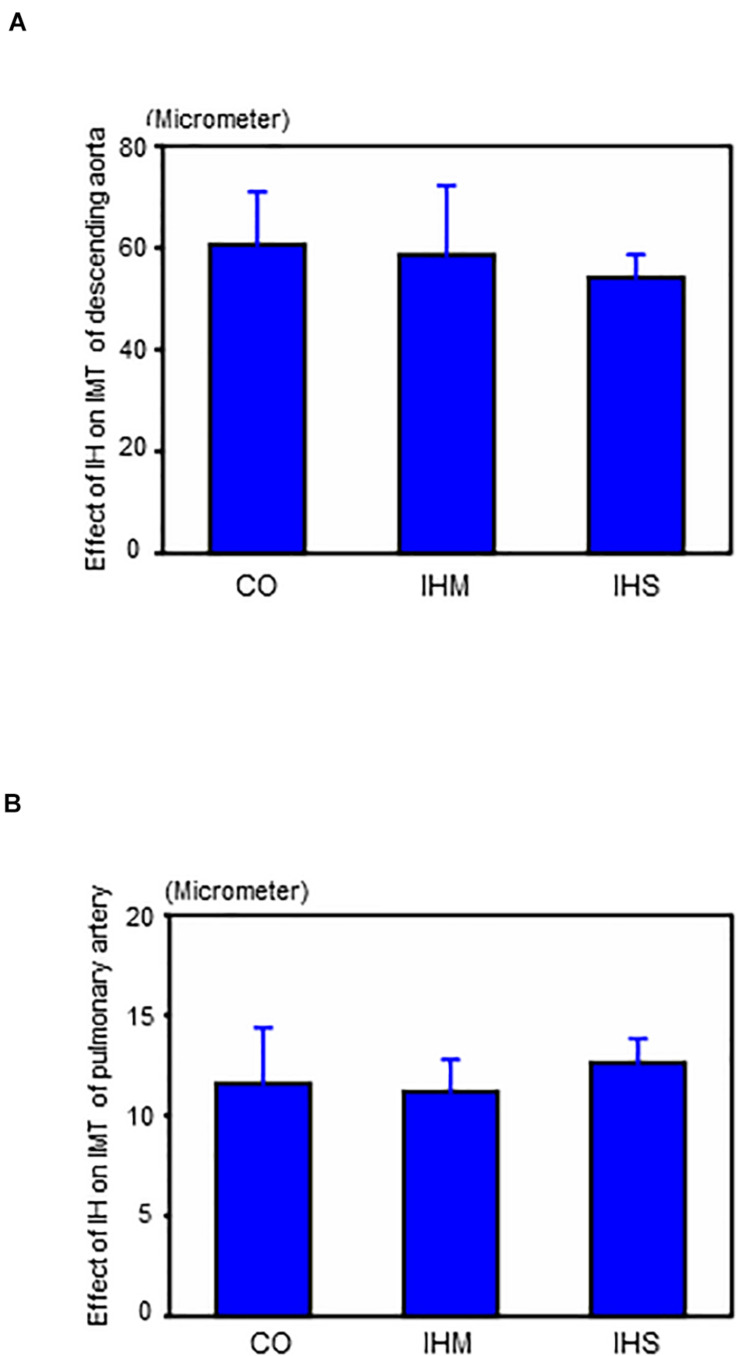
Effects of intermittent hypoxia in the normoxic recovery phase. **(A)** Effect of intermittent hypoxia (IH) on intima-media thickness (IMT) of descending aorta. **(B)** Effect of IH on IMT of pulmonary artery. Bars: SEM. CO, control; IHM, mild intermittent hypoxia; IHS, severe intermittent hypoxia. Neither IHS nor IHM induced significant changes in IMT of aorta or pulmonary artery in the normoxic recovery phase. ANOVA with Fisher’s *post hoc* test.

When compared to matched controls, we found no evidence that either IHS or IHM induced significant changes in IMT of aorta or PA after completing the 5-week normoxic recovery phase (Figures 2, 3). Persistent disruptions of the elastic laminae were not apparent in either the aorta or PA (Figure 2), suggesting that if such lesions were induced by IH, they had recovered at the end of the normoxic period.

### Evaluation With Review Managing Software

**FIGURE 4:**
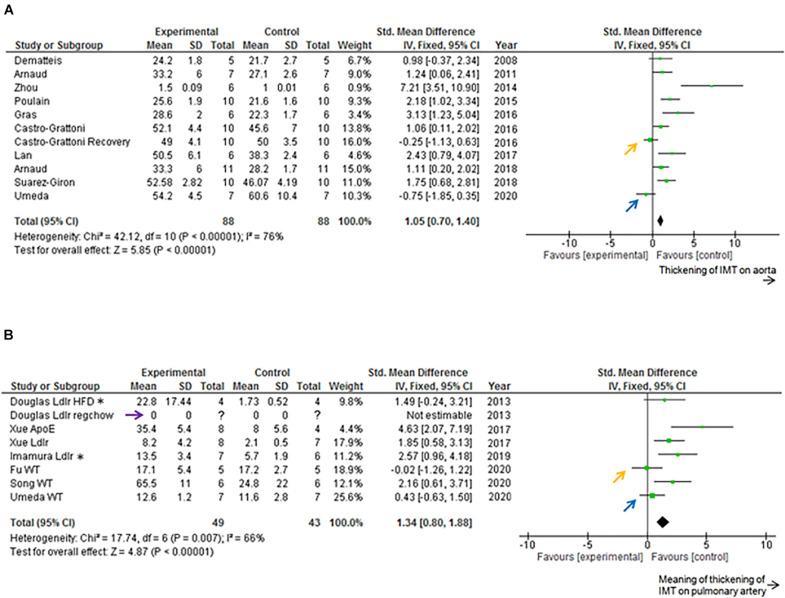
Forest plot. **(A)** Effect of intermittent hypoxia (IH) on intima-media thickness (IMT) of descending aorta. IH significantly increased aortic IMT (*P* < 0.01). Castro-Grattoni et al. (2016) examined both the effects of IH and those after 6 weeks of normoxic recovery (orange arrow). Current data are those after 5 weeks of normoxic recovery (blue arrow). Only 4–5 per condition were used by Dematteis et al. (2008) and Gras et al. (2016) used 5–6 mice per condition, and Arnaud et al. (2018) employed 9–13 mice per condition. All the experiments were performed on wild-type mice. **(B)** Effect of IH on IMT of pulmonary artery (PA). IH significantly increased IMT-associated parameters of PA (*P* < 0.01). Douglas et al. (2013) used both regular chow and high fat diet, and reported that regular chow did not cause atherosclerotic lesions of PA (purple arrow). Xue et al. (2017) and Imamura et al. (2019) used high fat diets. Douglas et al. (2013); Xue et al. (2017), and Imamura et al. (2019) used transgenic mice. Fu et al. (2020) reported that IH did not increase IMT of PA (orange arrow). Current data after normoxic recovery phase (blue arrow). ApoE, ApoE knockout mice; CI, confidence interval; HFD, high fat diet; IV, inverse variance-weighted method; Ldlr, Ldlr knockout mice; regchow, regular chow; SD, standard deviation; WT, wild-type mice. *Data of 8 week, 10 h/d exposure to Ldlr knockout mice.

**FIGURE 5:**
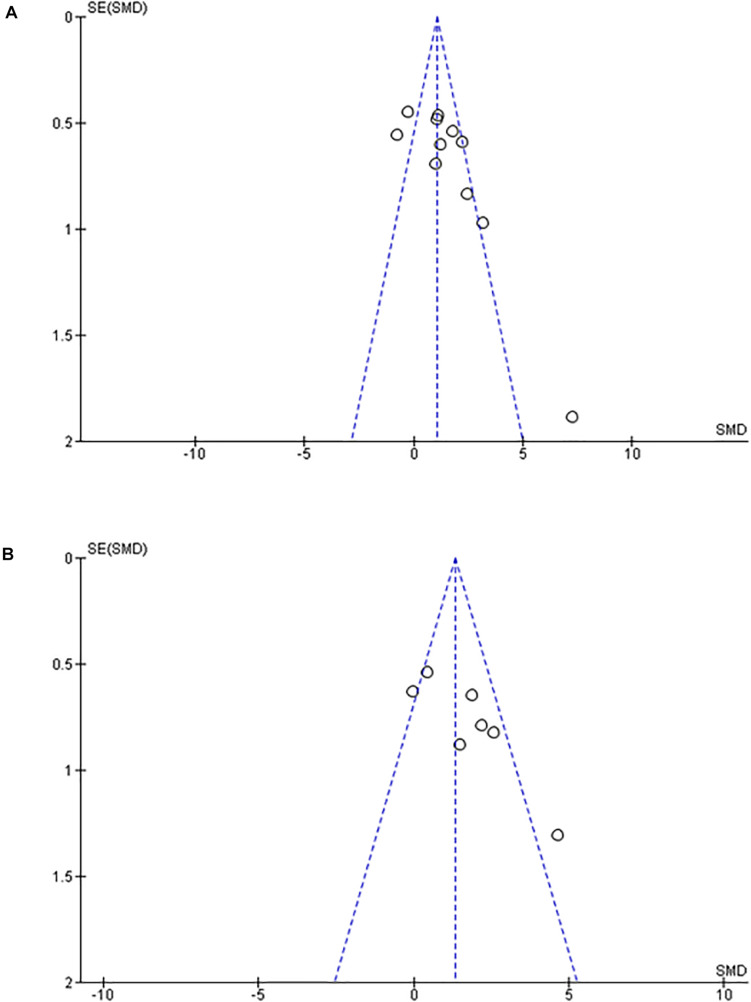
Funnel plot. **(A)** Effect of intermittent hypoxia on intima-media thickness of aorta. **(B)** Effect of intermittent hypoxia on intima-media thickness of pulmonary artery. Scarceness of plots in left lower area emerged for both aorta **(A)** and pulmonary artery **(B)**. SE, standard error; SMD, standardized mean difference.

Data from previous studies and current data are included in Figure 4 (Forest Plot) and Figure 5 (Funnel Plot). Most of previous studies focused on the aorta showed statistically significant increases in the IMT values following chronic IH (Figure 4A). The study by Castro-Grattoni et al. (2016) is the only one that examined IMT changes immediately upon completion of the 6-week exposure to IH and following a normoxic recovery phase of equivalent duration. Both that study and our current experimental data reveal that normoxic recovery is accompanied by reversal and normalization of IH-induced IMT increases in aorta (Figure 4A and Table 1).

Previous studies focused on PA showed statistically significant increases induced by chronic IH on PA IMT or atherosclerosis lesion area (%) data on the luminal surface of PA (Figure 4B). We could not find any previous studies examining the normoxic recovery phase for PA (Table 2). From our experimental results, IMT values in the normoxic recovery phase were smaller than those obtained just after IH for both aorta and PA, and such recovery values were similar to control conditions.

Of note, in the Funnel Plot, fewer data in the left lower area than in the right lower area were apparent for both aorta and PA (Figures 5A,B).

## Discussion

The current study adds incremental inferential data that suggests that following chronic IH exposures mimicking OSA of relative short duration (6–8 weeks), normoxic recovery is associated with normalization of the IMT in both aorta and PA. Review of previous publications reporting findings on IH exposures in mice, and the effects on IMT enabled us to proceed with unbiased comparisons of their findings with those of our experimental data. More importantly, the unbiased review revealed that evidence related to recovery of the effects of IH on either the aorta or PA is markedly scarce, and therefore deserves to be expanded in future studies. Notably, the Funnel plot graphs for both aorta and PA revealed a potential “publication bias,” and therefore further reinforce the need for incremental studies that should possibly include longer exposure durations as well as longer recovery periods (Cortese et al., 2017; Trzepizur et al., 2018). Indeed, one-sided scarce plots in the lower area in Funnel plot graphs are suggestive of publication biases. Thus, use of the RevMan software was particularly helpful for interpretation of our new experimental data with previously published findings in an unbiased fashion. Indeed, considering the scarcity of articles investigating the effects of IH on PA IMT, it is likely that IMT thickening may occur on aorta, but it is less likely to occur on PA. However, Xue et al. (2017) reported that PA atherosclerosis occurred in both ApoE knockout mice and Ldlr knockout mice, while aortic atherosclerosis occurred only in ApoE knockout mice. Based on the current results and the potential publication biases inherent to the available datasets, we conclude that the evidence indicating that IH induces IMT thickening of either the aorta or PA is less than definitive and requires extensive future exploration to either confirm or refute such assumptions.

Prior to our current experimental data, we found only two articles exploring normoxic recovery after IH exposures (Castro-Grattoni et al., 2016; Cortese et al., 2017), and only one of these articles evaluated IMT in the mouse aorta (Castro-Grattoni et al., 2016). These investigators showed that after 6 week-exposures to IH there was a complete recovery from IH-induced IMT thickening of the aorta by exposures to normoxia for additional 6 weeks. However, when the IH exposures were lengthened to 12 weeks, i.e., potentially more relevant durations as related to OSA (most patients with OSA are usually diagnosed many months or years after symptom onset), persistent disruption of the elastic laminae and ongoing pro-inflammatory signaling of aorta vascular wall macrophages was detected, suggesting that the reversibility of IH-induced vascular changes may progressively be attenuated as the duration of IH is increasingly prolonged (Cortese et al., 2017). This issue if obviously of great importance in light of the recent negative multicenter trials, whereby patients with OSA treated with CPAP did not derive specific cardiovascular benefits from the therapy (McEvoy et al., 2016; Labarca et al., 2020; Sánchez-de-la-Torre et al., 2020). Other murine studies have also shown either incomplete or time-dependent functional recovery following IH-induced deficits. For example, 12-week IH exposures induced significant insulin resistance and white adipose tissue inflammation in mice that were only partially reversed after a 6-week normoxic recovery (Gileles-Hillel et al., 2017). Furthermore, Gozal et al. (2017) showed that as the duration of IH increased, the probability of cognitive function recovery was reduced. Thus, on the one hand there appear to be exposure duration, mouse strain, and hypoxia severity dependencies that may dictate the potential emergence of specific vascular phenotypes, and on the other, the reversibility of such putative changes may be dependent of the same antecedent determinants of the vascular changes.

Meyrick and Reid. (1980) demonstrated that sustained hypoxia of 380 torr for 10 days caused thickening of PA media in rats and 70 days of normoxic recovery reduced the medial thickness to within normal range. However, we could not find any articles on the time course of chronic IH-induced IMT thickening of PA and its reversibility during a normoxic recovery phase in mice. Therefore, our findings suggest for the first time the possibility that such changes, if indeed induced by IH, are reversible under normoxia. Inferential clinical evidence would suggest that treatment with CPAP is accompanied by improved pulmonary hemodynamics in patients with severe OSA (Laks, 1995; Sajkov et al., 2002; Lattimore et al., 2008). However, critical review of the evidence casts significant doubt as to whether OSA indeed directly induced pulmonary vascular changes that manifest as pulmonary hypertension, or whether the latter is the result of left heart dysfunction (Ismail et al., 2015; Kholdani et al., 2015; Wong et al., 2017).

We took advantage of existing specimens from our previous experiments, in which energy expenditure and body weight changes were evaluated during the normoxic recovery phase in mice (Umeda et al., 2020). Specimens were fixed after the exposure experiments were completed. Therefore, limitations of the current study are that histopathological specimens immediately after cessation of IH were not available. Such specimens would better illustrate the IMT changes induced by IH, and therefore lend further reinforcement to the reversal upon return to normoxia.

In summary, our original assumptions that chronic IH would lead to sustained aortic atherosclerosis and PA thickening were not confirmed, since following normoxic recovery after chronic IH of different severities the IMT values were within normative control ranges. Careful review of the extant literature reveals a high likelihood of publication bias on this particular issue, and therefore well-designed studies examining both the effects of IH on aorta and PA IMT and the effect of normoxic recovery as a correlate of OSA treatment are urgently needed. OSA may induce IMT thickening (e.g., aorta and/or PA), but adherent treatment (e.g., nasal CPAP) will improve it.

## Data Availability Statement

All datasets generated for this study are included in the article/supplementary material.

## Ethics Statement

The animal study was reviewed and approved by the institution animal experiment ethical committee approved the protocol (International University of Health and Welfare animal experiment ethic committee approval number: 18020).

## Author Contributions

AU, YO, and DG: study design. AU, KM, AM, and HT: animal experiments and data collection. KT: making of IH system. AU: data analysis. All authors: interpretation, drafting, and approving of the manuscript.

## Conflict of Interest

The authors declare that the research was conducted in the absence of any commercial or financial relationships that could be construed as a potential conflict of interest.

## Footnotes

1 https://training.cochrane.org/online-learning/core-software-cochrane-reviews/revman/revman-5-download
